# Supplementary material for: From viability to cell death: Claims with insufficient evidence in high-impact cell culture studies
Source: PLoS One. 2022 Feb 22;17(2):e0250754. doi: 10.1371/journal.pone.0250754 (PMC8863264; doi:10.1371/journal.pone.0250754)
Supplement: Attachment — Submitted filename: renamed_ab46f.docx [file pone.0250754.s002.docx]

**COMMENTS – Manuscript PONE-D-21-12647**

Title: “Evidence provided by high-impact cell culture studies does not support authors' claims”

**Important note: This review pertains only to ‘statistical aspects’ of the study and so** **‘clinical aspects’ [like medical importance, relevance of the study,** **‘clinical significance and implication(s)’ of the whole study, etc.] are to be evaluated [should be assessed] separately/independently. Further please note that any ‘statistical review’ is generally done under the assumption that (such) study specific methodological [as well as execution] issues are perfectly taken care of by the investigator(s). This review is not an exception to that and so does not cover clinical aspects {however, seldom comments are made only if those issues are intimately / scientifically related & intermingle with ‘statistical aspects’ of the study}. Agreed that ‘statistical methods’ are used as just tools here, however, they are vital part of methodology [and so should be given due importance].**

**COMMENTS:** In my opinion, this authors group [now there are only two but may include few more similar minded scientists working in same field] can very-well start developing ‘publishing guidelines’ for such articles/literature {I guess, that will be very useful (cell culture studies will likely support authors’ claims subsequently) and definitely will be a great contribution}. It seems that OECD’s ‘Guidance Document on Good In Vitro Method Practices (GIVIMP)’ is not sufficient. Is not that true? There are only nine articles quoted [included in reference, may be because only these many are available] but are good (very relevant) articles. Congratulations.

For this purpose {developing ‘publishing guidelines’ for such articles/literature }, excellent article quoted as reference-1, [Ioannidis JPA. Why Most Published Research Findings Are False. PLOS Med. 2005;2: e124. 260 doi:10.1371/journal.pmed.0020124] may be useful. The understudied aspect of this quality issue in published work highlighted is “the harmony between the hypotheses and the experimental design” is true for many areas and is appreciable. My only question is (line 52) ‘controls (positive, negative)’ {i.e. what are ‘positive controls and negative controls?}. Please clarify. Little/brief clarification of terms ‘p-hacking and cherry picking’ [like ‘P-hacking’ is the relentless analysis of data with an intent to obtain a statistically significant result, usually to support the researcher’s hypothesis and ‘Cherry-picking’ is the presentation of favourable evidence with the concealment of unfavourable evidence] could be useful for readers, I guess.

Although it is not my ‘Subject Area’, in my opinion, this one it is an excellent article. Nevertheless, I feel that overall the article is little short. Maybe adding few more case studies will do. As pointed out in ‘important note’ above “This review pertains only to ‘statistical aspects’ of the study and so ‘clinical aspects’ should be assessed separately/independently. May think of changing title (may make it more catchy).
