# Supplementary material for: From viability to cell death: Claims with insufficient evidence in high-impact cell culture studies
Source: PLoS One. 2022 Feb 22;17(2):e0250754. doi: 10.1371/journal.pone.0250754 (PMC8863264; doi:10.1371/journal.pone.0250754)
Supplement: Attachment — Submitted filename: Ozkaya Response to Reviewers.docx [file pone.0250754.s003.docx]

Authors: We would like to thank all reviewers for their constructive criticism.

Reviewer #1

Authors: We would like to thank the reviewer for encouraging comments. There is indeed a lack of a comprehensive guideline for basic cell culture research especially regarding simple assays and how to interpret their results. I hope we can eventually start developing such guideline. We will seek likeminded researchers for this purpose as the reviewer has suggested.

Specific points:

R1: My only question is (line 52) ‘controls (positive, negative)’ {i.e. what are ‘positive controls and negative controls?}. Please clarify. Little/brief clarification of terms ‘p-hacking and cherry picking’ [like ‘P-hacking’ is the relentless analysis of data with an intent to obtain a statistically significant result, usually to support the researcher’s hypothesis and ‘Cherry-picking’ is the presentation of favourable evidence with the concealment of unfavourable evidence] could be useful for readers, I guess.

Authors: An explanation has been added for positive and negative controls (lines 53, 54) as well as p-hacking and cherry picking (lines 56, 57).

R1: May think of changing title (may make it more catchy).

We did try to find a catchier title when we first wrote the manuscript, but we did not like the titles we came up with.

Reviewer #2

Authors: Thank you for the reviewer’s comments and support. We tried to be as transparent and as clear as possible when writing the manuscript. Therefore, we really appreciate that our efforts did not go unnoticed.

Specific points

R2: 1) It would be helpful for the Authors to clarify their intent. Specifically, it is not clear to this Reader whether the Authors are stating that 64% of studies were supported with sufficient evidence, or 64% were not supported by sufficient evidence. The two lines that appear to be in conflict are: Line 33: “Our findings revealed that only 64% of all claims were sufficiently supported by evidence” versus Line 195: “we considered the evidence …which was asserted by 66 different studies (64%), as insufficient”

Authors: Those statements are not in conflict, but we can understand the confusion. The first one was the ratio of sufficient claims (180/282). Second one was the ratio of studies with insufficient evidence (66/103). The number being same is just a coincidence. However, during revision we made minor changes in the analysis considering the feedback from the reviewers altering claim sufficiency rate to 63% (178/281) (line: 33). The number of studies with insufficient evidence stayed the same (line: 207)

2) Two areas that were not specifically addressed, but that could further strengthen the manuscript are: a) The Authors did not mention that subjective analyses should be performed by blinded investigators. Because it is seldom the case that these type of experiments are performed by blinded researchers, it would be valuable for the Authors to highlight its importance.
b) When considering an effect on cell proliferation or cell death, the Authors should stipulate that cell proliferation or cell death should be expressed on a logarithmic not a linear axis. Clearly cell proliferation is an exponential, not a linear function. Although many papers misrepresent their data by presenting it on a linear axis, where it looks more dramatic, this is not an appropriate way to present the data. Given the focus of this publication, this too would be a valuable addition for the Authors to comment upon.

Authors: We added a section containing relevant information to the discussion (lines: 280+). We included the importance of blind investigators as well as the importance of logarithmic scale for proliferation studies.

R2: Minor issues: Line 162: Blc-2 should be Bcl-2

Authors: Corrected.

Reviewer #3

Authors: Thank you for your valuable suggestions. We hope that the article is now more rigorous after this revision.

Specific points:

R3: 1. Line 54 – is this substantiated by any references, or anecdotal?

Authors: The references are listed in the previous sentence. Especially by the work of Ioannidis and his colleagues. We originally did not want to repeat the same references in two consecutive sentences. However, in accordance with the comments, we added them to the second sentence as well.

R3: 2. The introduction and overall manuscript is lacking references

Authors. Manuscript was revised and new references were added.

R3: 3. Lines 67+ reads more like commentary – also a bit vague. What is being discussed here? The cytotoxicity and cell viability terms?

Authors: The idea of this study emerged thanks to one simple observation: there were studies using tetrazolium reduction assay results to justify different claims such as viability, cytotoxicity etc. The core of this work is about whether these “claims” are properly sported by “evidence”. Therefore, we tried to clearly define terms corresponding to different claims. For this purpose, we first searched the literature, utilized the OECD guide and finally wrote our own definition. We believe it is important for our readers to understand our process clearly and fully. That is why that section was added.

R3: 4. What was the rationale for focusing on 2017 and 2018?

Authors: We began our analysis in March 2020. We wanted to include a two-year period, we wanted to give the articles one year to receive citations (therefore we did not investigate 2018-2019) and we wanted to focus on the most recent work.

R3: 5. The inclusion/exclusion should be detailed more clearly on lines 91+

Authors: We re-wrote the part.

R3: 6. Since care is being taken to describe these cell health variables… viability I find a bit problematic. It is not the number of living cells, it generally represents a reaction that is often considered to have a linear relationship to the number of living cells. These enzymes, such as tetrazolium salt, etc., can be modified by exogenous factors and cannot be explicitly considered to describe living cell number.

Authors: We agree with the reviewer on the issue. Metabolic activity is not a very good indicator of cell viability. This is especially problematic when your treatment also modifies cellular metabolism. Imagine that you have developed a drug targeting mitochondrial metabolism. It is clear that you should not use tetrazolium reduction assay to evaluate cell viability. However, there are many studies doing exactly that. That is why it is important to address these issues. The same thing can be said for membrane integrity being an indicator of cell death, caspase-3 activation an indicator of apoptosis etc. However, in this study we did not want to focus on that because: even though tetrazolium reduction assay may not be an accurate indicator of viability, it is most definitely not an indicator of apoptosis. Yet there are highly-cited articles claiming just that. They say that the treatment induced apoptosis by providing only MTT results as evidence. This was the clearer and the bigger problem and we focused on that. However, we do take the comments of the reviewers into account and therefore we added a section to the discussion focusing on the problems pointed out (lines: 280+).

R3: 7. Line 144 – LDH release is a cell viability assay, not a cytotoxicity assay – given the context of this paper, this is a very important point to classify these assays correctly. There are a variety of contexts described in the literature where cells leak LDH and remain otherwise alive and will respond normally via other cell viability measures. Adipocytes is one example of this, though others have been described. Unless the specific experimental context is being taken into account, it’s hard to see how these sorts of generalities are not going to adversely influence results here.

Authors: Literature may seem to disagree with the reviewer on this (1-3). LDH assay almost always has been used to measure cytotoxicity. However, we agree that we should question validity of using membrane integrity as a measure of cell death.

References:

1. An enzyme-release assay for natural cytotoxicity. C Korzeniewski, D M Callewaert. 1983
2. Validation of an LDH assay for assessing nanoparticle toxicity. Xianglu Han et al. 2011.
3. Cytotoxicity Assays: In Vitro Methods to Measure Dead Cells. Terry Riss et al. 2019

R3: 8. Table 1 – how is DNA content not included in cytotoxicity? It is the gold standard for determining cell number. You could say that it is context dependent, if there are no wash steps in a particular assay, but it cannot just be ignored as a cytotoxicity measure.

Authors: Table 1 was re-constructed. We did not form the table from out understanding of these assays and the evidence they provide. We formed the table from the data we obtained from the studies we have investigated. Therefore, if there is no study utilizing DNA content as a measure of cytotoxicity we cannot have it there. It is not a general statement; it is just a re-organization of the data we obtained from our analyses. However, since table was criticized for being not clear enough, we created another table and include a more detailed explanation to how we have analyzed the studies and created the table.

R3: 9. 9. Given the well-described artifacts and issues with a lot of the cell viability and proliferation assays, as described by Hsieh et al., 2015, 2016, etc., this article seems to ignore many of these issues with the assays they included.

Authors: We included one of the studies as the reviewer suggested.

R3: 10. There doesn’t seem to be any transparency about how and why individual publications were deemed sufficient or insufficient. If highly cited publications are going to be published as insufficient, a rationale column for the decision-making process is critical to providing clarity for readers.

Authors: We re-constructed table 1 to draw a clearer picture of our evaluation process. We included a rationale column as the reviewer suggested.

R3: 11. Lines 208+ - cytotoxicity is not a measure of viability, it is a different measure completely.

Authors: We completely agree on cytotoxicity not being a measure of viability. However, we encountered articles using cytotoxicity as if it represents viability during our investigation. We included that section to acknowledge it. We do not defend it.

R3: 12. Line 206 – “we had to make assumptions on behalf of the researchers” is not very scientific. In a systematic review context, you can and should reach out to the authors for clarification. Appreciate this is not that, but if you are publishing a paper blasting the authors for having an “insufficient” study, and only rely on your interpretation, that is a problem.

R3: 13. Moreover, interpretation of who? Is this a single author performing these assumptions? Is each paper reviewed by both authors independently? A single person evaluating these is not robust – and what sort of testing was done to verify that the authors performed these assumptions in a consistent manner?

Authors: There is no assumption and interpretation in the analysis part. The assumptions /interpretations were general and were carried out them before the analysis. The assumptions are listed as definitions in the materials and methods section. When we define viability, cytotoxicity, proliferation etc. we “assume” they the authors of the articles we are analyzing have used it the same way. But since we cannot know it for sure we included the section previously discussed (lines 217+)

We agree that out evaluation process was not clear enough in the original manuscript. We re-constructed Table 1 and included more text to clarify our process.

R3: 14. I feel at this point in the paper that I’m not sure of the overall goal. Are the authors just using the assay reported in the paper and checking which box on a table it fits into? This doesn’t seem like the level of discourse that is particularly worthy of a peer-reviewed publication. A commentary could fit this, but it seems more opinion than a rigorous evaluation of reproducibility – which is, of course, crucially important.

Authors: We included more text to clarify our process. We tried out best to make the analysis as objective as possible. This study is not an evaluation of reproducibility. We focused on the claims and the evidence of the articles we have investigated. This study is neither a commentary nor a review. We had a hypothesis “highly cited articles do not have sufficient evidence to support their cell culture-related claims” and we focused on selected claims developed a strategy to test the hypothesis. We gathered data, made our analysis and have reached a conclusion.

R3: 15. The first sentence of the discussion is very vague and alarmist – the only thing examined here was the cell proliferation/toxicity/viability and not any other claims these publications may have made. That is very important to make clear

Authors: The discussion was revised.

Reviewer #3

Authors: We would like to thank the reviewer for helpful comments that substantially improved the quality of this manuscript. We hope that it could be considered for publication at its current stage.

R4: Add a citation to papers that have addressed similar issues – line 56-57.

Authors: Citations added.

R4: Fig. 1C dehydrogenase activity is always used correctly to assess viability?

Authors: That is correct. In this study we considered dehydrogenase activity measurement to provide sufficient evidence for viability independent from the experimental design. On the other hand, we are well aware of metabolic activity not being a very accurate indicator of cell viability. We included a section in discussion to discuss it (lines 280+).

R4: 3. Fig. 1 C (discussed on lines 219-220) you say data are interpreted correctly in 73 claims, but the figure looks like it should be 48 claims. Please correct.

Authors: Corrected.

R4: 4. Thorough copy-editing is needed

Authors: Manuscript was revised accordingly.

R4: 5. Nomenclature recommendations should not be buried in the Methods. You highlight this as a main conclusion of your work. There is an opportunity to be prescriptive with this work and for it to be a resource for people to turn to when selecting assays and designing experiments – I think this should be focused on much more.

Authors: That was our original intention. However, we realized that we needed those definitions in the materials and methods section to clearly explain our process. Considering the reviewers’ comments, we also included the definitions to discussion section and referred to them in the abstract.

R4: 6. Your definition of proliferation rate is presented as a population-level readout “how fast a group of cells divide over time” however it is then contradicted by stating that this rate only applies to surviving cells – an apparent decrease in proliferation rate of a population could occur from a slowing of the doubling time of all cells in a population or from a fractional response in which a subpopulation is killed off, and the surviving cells continue to proliferate at the same rate (or at an altered rate) resulting in a longer apparent doubling time, as you point out. Is your proposed definition of proliferation rate meant to be applied to single cell or population level data? Does it only apply to living cells at endpoint? If the endpoint is simply how fast a group of cells divide over time, then it doesn’t matter if the underlying phenotypes are different, though they should be determined with secondary assays designed specifically to measure cell death and growth rate.

Authors: The main point is to differentiate slowing of the doubling time from killing a sub-population. We embraced that distinction to differentiate “proliferation rate” from “viability” and to give it a unique identity. We agree that the best way to measure it is to measure growth rate along with the cell death and we debated to decide what would be an acceptable evidence to demonstrate proliferation rate independent from cell death. Beside the usual markers which were specifically designed to measure proliferation rate, we decided “if there is no apparent reason for cell death (i.e. no treatment) and cells are being observed over time to observe how fast a group of cells divide over time, viability change can indicate proliferation”. However, it is important to note that while measuring proliferation-specific one must also consider the impact of cell loss due to death similar to the case with the viability. In essence, if cells die via cell death you may end up reading lower levels of Ki67 expression or nucleotide incorporation. That is why the proliferation data should also be normalized based on living cells. We included that recommendation to the discussion (lines: 280+).

R4: 7. I strongly disagree that measuring metabolic activity is sufficient for assaying cell viability. In some cases, these techniques (MTT, CellTiter-Glo, etc.) yield results that correlate linearly with live cell count, but many perturbations directly affect the metabolic activity of cells which can result in critical miss-interpretations of effects. See https://pubmed.ncbi.nlm.nih.gov/31302153/ for clear examples.

We agree with the reviewer. We do not know how accurately metabolic activity indicates cell viability. This is especially problematic when your treatment also modifies cellular metabolism. Imagine that you have developed a drug targeting mitochondrial metabolism. It is clear that you should not use tetrazolium reduction assay to evaluate cell viability. However, there are many studies doing exactly that. That is why it is important to address these issues. The same thing can be said for membrane integrity being an indicator of cell death, caspase-3 activation an indicator of apoptosis etc. However, in this study we did not want to focus on that because: even though tetrazolium reduction assay may not be an accurate indicator of viability, it is most definitely not an indicator of apoptosis. Yet there are highly-cited articles claiming just that. They say that the treatment induced apoptosis by providing only MTT results as evidence. This was the clearer and the bigger problem and we focused on that. However, we do take the comments of the reviewers into account and therefore we added a section to the discussion focusing on the problems pointed out (lines: 280+).

R4: 8. With respect to defining cell death, I generally agree that this needs to be measured directly, however, if the number of cells are counted at the time of drug addition, for example, then a treatment resulting in net cell loss can only result from some degree of cell death. In general, any time cells are counted, live and dead cells should be distinguished at the bare minimum. See Hafner et al. (https://pubmed.ncbi.nlm.nih.gov/27135972/) for a more nuanced and thorough discussion. Growth rate inhibition metrics, an important addition to the field, are omitted and should be discussed – they are immediately relevant and address several of the same concerns the authors highlight.

Authors: We included discussion of these metrics (lines 270+).

R4: 9. Apoptosis, please correct typo in “molecular switch responsible in apoptosis” to “molecular switch resulting in apoptosis” and specify whether you’re referring to cleaved caspases and/or MOMP.

Authors: Corrected

R4: 10. Please clarify why you don’t consider PI sufficient to assay membrane integrity but you do consider other dyes that are also membrane impermeable to be sufficient (lines 174-177) viability assays.

Authors: Thanks to the comments of the reviewer, we realized that we made a mistake evaluating the use of membrane integrity markers for measurement of viability. We now classify PI and AAD7 as insufficient. We only accepted trypan blue as sufficient because living cells can be detected via trypan blue assay. We re-calculated all the numbers and re-draw all the graphs accordingly.

R4: 11. Table 1 as is, is confusing especially for the proliferation and viability entries. It would be much more useful if it was presented by claim and then assay, followed by what each assay actually measures, and what is required for that assay to be sufficient for that claim.

Authors: Table-1 is reconstructed to better reflect our evaluation process.

R4: 12. Only nine references are provided – there is a lot of literature that has been overlooked on assay choices and pitfalls that should be considered, and incorporated into the introduction and discussion sections.

Authors: More references are added.
