# Supplementary material for: From viability to cell death: Claims with insufficient evidence in high-impact cell culture studies
Source: PLoS One. 2022 Feb 22;17(2):e0250754. doi: 10.1371/journal.pone.0250754 (PMC8863264; doi:10.1371/journal.pone.0250754)
Supplement: Attachment — Submitted filename: PLOS ONE R1 review.docx [file pone.0250754.s004.docx]

The revised manuscript “Evidence provided by high-impact cell culture studies does not support authors’ claims” from Ozkaya and Geyik is improved from the initial submission; however, several points still need to be addressed and clarified prior to publication. The expansion of Table 1 is a significant improvement to the previous version.

Minor points:

1. Table 1: I think it would be more intuitive to change the order slightly so the cell death claims (apoptosis, cell death, cytotoxicity) are followed by the live cell claims together (cell growth, proliferation, viability) rather than strictly listing them alphabetically.
2. Thorough copy editing is still required. Much of the text remains unchanged.
3. Referring to cell state assays as ‘cell culture techniques’ (for example on lines 41, 266 and 282) is confusing. I perceive good cell culture technique to be the proper maintenance of cells in culture (free of mycoplasma and other contaminants, log-phase growth for the assays you discuss etc). Good cell culture technique is also a critical factor in generating high quality and reproducible measures of cell viability/death/proliferation etc. It would be worth adding that all of the assays discussed can be badly confounded by poor cell culture technique. As you correctly point out, cost, simplicity, speed, and throughput likely motivate assay selection.
4. Reference to Fig 1D (line 226) should be to 1C.
5. While a valuable clinical biomarker, ki67 positivity as it relates to cell cycle and cells cycling is complicated in culture systems, and many cells in arrested states remain ki67 positive – caution in its interpretation is warranted, and like most of the endpoints discussed should be cross-validated. See <https://www.ncbi.nlm.nih.gov/pmc/articles/PMC6108547/> and <https://elifesciences.org/articles/13722>
6. Lines 37-38 are unclear.
7. Consider reframing line 76 from ‘we decided to define these terms ourselves’ to ‘in the current work, we propose a series of definitions and recommendations’ since this is the ultimate goal.
8. Lines 132 and 133, please be specific about the ‘morphological changes’ and ‘molecular switches’ that you are referring to.
9. Please add the citations for id#9 and id#26 in the text (lines 260 and 261) so the reader can refer to them directly without having to access your web tool.
10. Line 271: GR refers to normalized growth rate inhibition, not simply growth rate.
11. Line 272: DIP should be specified ‘drug-induced proliferation rate’
12. Lines 273-276 are unclear. Please explain.

Major points:

1. Your claim in Table 1 that ‘DH activity correlates with the number of viable cells’ is not always true; at the very least, the associated ‘Yes’ should be changed to ‘Conditional’. Moreover, you twice assert that you consider measurements of metabolic activity as sufficient evidence for viability because they’re widely used: ‘…it is the most common method used to evaluate viability…’ (line 119) and ‘…we considered measurement of metabolic activity as an indicator of viability as it is by far the most preferred viability assay…’ (lines 283-284). This is poor justification for reasons you include in the discussion and reasons brought up in the previous reviews. With proper controls/assay calibration (how does the assay output relate to live cell count in the specific context?) and cross-validation experiments to confirm that metabolic activity is not influenced by the perturbation under study, these assays of metabolic activity can be used as surrogates for viability, but these controls are critical to correct data interpretation. My concern is that these points are not sufficiently addressed, and that incorrect use of metabolic assays to evaluate cell viability and proliferation is presented as acceptable without caveats throughout the body of the paper only to be contradicted in the discussion.
2. Can you comment on how often, in the papers you included, the assays you considered are the *only* line of evidence for a conclusion drawn since this seems to be the most problematic case? A common scenario is one where a crude, population level measure such as MTT is used to suggest toxicity, and then followed by a microscopy or flow cytometry-based assay to confirm cell death vs change in proliferation and mechanism (apoptosis vs necrosis etc). In a case like that, would the use of a metabolic assay be considered sufficient or insufficient? The use of complimentary and confirmatory assays should be discussed and considered when scoring claims made in the papers that you evaluated.
