# Supplementary material for: From viability to cell death: Claims with insufficient evidence in high-impact cell culture studies
Source: PLoS One. 2022 Feb 22;17(2):e0250754. doi: 10.1371/journal.pone.0250754 (PMC8863264; doi:10.1371/journal.pone.0250754)
Supplement: Attachment — Submitted filename: Ozkaya 2nd Response to Reviewers.docx [file pone.0250754.s005.docx]

Please specially address the concerns raised by reviewers 3 and 4. It might be helpful, if you can revise the title of the manuscript, so it is more specific ad does not seem dismissive towards all "authors' claims". Also, the text could also be slightly revised to clarify that the objective is not to dismiss all the findings these publications are reporting.

The title was changed, and we included several statements to different sections of the article to clarify the points the editor made.

Evidence provided by high-impact cell culture studies does not support authors’ claims

**Reviewer #1: COMMENTS: As already said, that everything [like execution, methodology, statistical analyses, etc] are very good [i.e. study is excellent]. Writing/presentation is also excellent. Later, comments made [probably by other respected reviewers as well] were/are attended or answered positively/adequately, I am fully satisfied and the manuscript is improved a lot. I recommend acceptance.**

Thank you.

**Reviewer #2: Thank you for addressing the comments that I had provided.**

**This is a useful contribution and an important contribution.**

Thank you.

**Reviewer #3: I appreciate the reviewer edits to the manuscript, though many of my original concerns remain. This article still reads to me as a commentary - an important topic for one - but is not well enough defined for publication as a research article.**

We believe that this work fits the definition of a “research article” embraced by PlosOne:

“Research Articles present the results of original research that *address a clearly defined research question* and *contribute to the body of academic knowledge*”

We have a clearly defined research question (are cell culture claims of high-impact publications sufficiently supported by experimental evidence), we have an experimental approach to test the hypothesis and obtained results. Finally, we believe that the findings of this study as well as our conclusions contribute to the body of academic knowledge.

We leave the final decision on the matter to the editor.

**I still have major concerns with the title of this article. The authors rather blindly call out other papers yet (Evidence provided by high-impact cell culture studies does not support authors' claims) yet they have not examined anything in these papers other than the use of cytotoxicity/cell viability assays. This absolutely must be made crystal clear. I asked for this on first revision and this was not addressed. The inflammatory title, when this is explicitly not assessed, is not proper.**

We have changed the title.

**It also needs to be explicitly stated that the claims they are examining from these papers are in some cases only a very small part of the overall paper. The authors in the response to reviewer document repeatedly state the rationale for this article, but they have strayed far beyond that in the paper itself, as written.**

We have changed the title and included statements to the abstract, introduction, and discussion sections to clarify this.

**I happen to disagree with the authors on the LDH assay, though appreciate there can be difference of opinion here. Though the irony of them citing the literature to defend correct interpretation is not lost on me, given the topic of their paper. I would suggest that they think about what this assay actually examines and whether that fits into the definition of a cell viability or toxicity assay. As there are clear examples where the cell type and context promote positive findings in this assay, it cannot be a cytotoxicity assay**.

After discussing the points made by the reviewer, we decided not to change our original position on the matter. Cytotoxic insult causes loss of membrane integrity and intracellular content such as LDH leak from death/dying cells. Measuring LDH activity in cell media correlates with the loss of membrane integrity thus, cytotoxicity. Like every indirect assay, it has its limitations, but those limitations are not enough to dismiss it.

It is important to note that the literature we cited in our previous response were carefully selected as they explain why membrane integrity was an indicator of cell death providing experimental evidence. We want to make it clear that even if we criticize published work in our study, we still believe citing published work is one of the pillars of science.

**Some language editing is still needed here, particularly for the newly added sections highlighted in yellow.**

Proofreading on manuscript was carried out by a native English speaker and changes were made accordingly.

**This article should be a much shorter commentary, where it could accomplish the author's goals and not over-reach.**

This has been answered in first point.

**Again, how is DNA content listed under proliferation and not toxicity? This was not appropriately responded to in revision**.

DNA content is listed under proliferation because that is how the articles we have investigated have used them. We did not decide which evidence supports which claims. That information was taken from the articles we have investigated. Since there was no article using DNA content to measure toxicity, we cannot have it in that list.

In other words, we can summarize the methodology used as:

1- Find the studies that contains at least one claim (such as cytotoxicity, proliferation, etc.)

2- Look for the methods used to make “that” claim in those articles (such as DNA content, dehydrogenase activity, etc.)

3- Decide whether the method used by the article provides sufficient evidence for the claim asserted in the article.

For example, when Study A claims cytotoxicity by using MTT, we included that to our analysis and decided whether it is sufficient or not.

Another example, when Study B claims proliferation by using DNA content, we included that to analysis and decided whether it is sufficient or not.

So, if any study among the investigated 103 studies have had claimed cytotoxicity by demonstrating DNA amount as evidence, we would have included that. And then we would have decided the sufficiency. However, there were no studies using DNA content as evidence for cytotoxicity.

By the way, we also agree with the reviewer on DNA content measurement being an appropriate technique to detect cytotoxicity, it just was not used in any of the 103 studies we analyzed and that is why it was not on the table.

**It is still unclear how some terms are being distinguished. On line 135, cytotoxicity, the definition specifically states "cell death". Yet in Table 1, cytotoxicity and cell death are separate groups on this table. I don't understand how these groups are being defined and how they are different, given the descriptions the authors are providing here**.

The table was generated by using the information gathered from the articles we have investigated. So, if the authors of an article used the term “cell death” or “cytotoxicity” in their manuscript, we included them separately in the table. Therefore, different claims in the table do not correspond to different definitions, that is just the data obtained from the articles. In fact, we considered cytotoxicity and cell death the same in our analysis. Similarly, we also considered growth and proliferation the same as well. However, we have written them separately in the table because the table contains the claims asserted by the authors of the articles we have investigated, and we just copied whatever they have chosen to write in their manuscripts. On line 135, on the other hand, we have written the definitions we have embraced in this study. That is why it specifically states “cell death” under cytotoxicity. We consider them as being equal and we carried out our analysis accordingly.

**Reviewer #4: PONE-D-21-12647_R1 review (also attached)**

**The revised manuscript “Evidence provided by high-impact cell culture studies does not support authors’ claims” from Ozkaya and Geyik is improved from the initial submission; however, several points still need to be addressed and clarified prior to publication. The expansion of Table 1 is a significant improvement to the previous version.**

**Minor points:**

**1. Table 1: I think it would be more intuitive to change the order slightly so the cell death claims (apoptosis, cell death, cytotoxicity) are followed by the live cell claims together (cell growth, proliferation, viability) rather than strictly listing them alphabetically.**

We have changed the order as recommended.

**2. Thorough copy editing is still required. Much of the text remains unchanged.**

Proofreading on manuscript was carried out by a native English speaker and changes were made accordingly.

**3. Referring to cell state assays as ‘cell culture techniques’ (for example on lines 41, 266 and 282) is confusing. I perceive good cell culture technique to be the proper maintenance of cells in culture (free of mycoplasma and other contaminants, log-phase growth for the assays you discuss etc). Good cell culture technique is also a critical factor in generating high quality and reproducible measures of cell viability/death/proliferation etc. It would be worth adding that all of the assays discussed can be badly confounded by poor cell culture technique. As you correctly point out, cost, simplicity, speed, and throughput likely motivate assay selection.**

Word “techniques” in mentioned lines was changed to “methods”.

**4. Reference to Fig 1D (line 226) should be to 1C.**

Thank you for the nice catch. We have corrected the figure reference.

**5. While a valuable clinical biomarker, ki67 positivity as it relates to cell cycle and cells cycling is complicated in culture systems, and many cells in arrested states remain ki67 positive – caution in its interpretation is warranted, and like most of the endpoints discussed should be cross-validated. See https://www.ncbi.nlm.nih.gov/pmc/articles/PMC6108547/ and** [**https://elifesciences.org/articles/13722**](https://elifesciences.org/articles/13722)

A part was added to the discussion to mention this.

**6. Lines 37-38 are unclear.**

The part was re-written.

**7. Consider reframing line 76 from ‘we decided to define these terms ourselves’ to ‘in the current work, we propose a series of definitions and recommendations’ since this is the ultimate goal.**

We have reframed the sentence as recommended.

**8. Lines 132 and 133, please be specific about the ‘morphological changes’ and ‘molecular switches’ that you are referring to.**

The part was specified.

**9. Please add the citations for id#9 and id#26 in the text (lines 260 and 261) so the reader can refer to them directly without having to access your web tool.**

Citations were added.

**10. Line 271: GR refers to normalized growth rate inhibition, not simply growth rate.**

It was corrected.

**11. Line 272: DIP should be specified ‘drug-induced proliferation rate’**

It was corrected.

**12. Lines 273-276 are unclear. Please explain.**

The part was re-written.

**Major points:**

**1. Your claim in Table 1 that ‘DH activity correlates with the number of viable cells’ is not always true; at the very least, the associated ‘Yes’ should be changed to ‘Conditional’. Moreover, you twice assert that you consider measurements of metabolic activity *as sufficient evidence for viability because they’re widely used: ‘…it is the most common method used to evaluate viability…’ (line 119) and ‘…we considered measurement of metabolic activity as an indicator of viability as it is by far the most preferred viability assay…’ (lines 283-284).* This is poor justification for reasons you include in the discussion and reasons brought up in the previous reviews. With proper controls/assay calibration (how does the assay output relate to live cell count in the specific context?) and cross-validation experiments to confirm that metabolic activity is not influenced by the perturbation under study, these assays of metabolic activity can be used as surrogates for viability, but these controls are critical to correct data interpretation. My concern is that these points are not sufficiently addressed, and that incorrect use of metabolic assays to evaluate cell viability and proliferation is presented as acceptable without caveats throughout the body of the paper only to be contradicted in the discussion.**

We agree with the reviewer, and we have made minor revisions to emphasize these points. However, after many discussions we decided not to change our analysis. Every assay has its limitations, and we must draw the line somewhere. In this study we decided to consider metabolic activity as an acceptable indicator of cell viability. The reviewer is correct on “being widely used” to be a poor justification. However, it is not only about being a commonly used tool. Viability measurement is the only purpose of an MTT assay that is somewhat acceptable. Again, it may not be perfect to measure viability, but the problem is that people are using it to measure cell death which is arguably far worse than using it to measure viability. And that is what we wanted to focus on this study.

We investigated randomly selected articles from our list to detect whether they have used controls to confirm effects on metabolism. None of the articles have done a proper assay calibration. Therefore, when we change the sufficiency of MTT from “yes” to “conditional”, it is instead going to be “no” for almost all the claims. We are simply not ready to consider MTT as insufficient evidence for viability.

Another issue with that approach was about the way this article presents its findings. We wanted to keep it as simple as possible to reach a wider audience. When we dive into the detail level in which we consider MTT as conditional evidence for viability, almost all experimental results are going to be “conditional evidence”. Indeed, proper controls and normalization are important for every assay, but that approach would turn this work into an article about cell culture assays and their weaknesses as we won’t be able to classify them as sufficient/insufficient for specific claims. We know that our current approach is also reductionist, but the idea is being not as reductionist as the work we criticize. By doing that we are hoping to reach more scientists and if we can make a couple of researchers question their decision to claim cell death with MTT data, we consider it as a win. After that initial step we can move on to a more complicated approach. We hope that someday we can be a part of team which creates a comprehensive guideline (on viability/cytotoxicity/proliferation rate etc) by which the current methodology is dissected, evaluated and practical recommendations are made.

**2. Can you comment on how often, in the papers you included, the assays you considered are the only line of evidence for a conclusion drawn since this seems to be the most problematic case? A common scenario is one where a crude, population level measure such as MTT is used to suggest toxicity, and then followed by a microscopy or flow cytometry-based assay to confirm cell death vs change in proliferation and mechanism (apoptosis vs necrosis etc). In a case like that, would the use of a metabolic assay be considered sufficient or insufficient? The use of complimentary and confirmatory assays should be discussed and considered when scoring claims made in the papers that you evaluated.**

When we were evaluating the articles’ claims we focused on the strongest evidence. So, if the article claimed apoptosis and has MTT and Annexin-V as evidence, we did not list MTT for their apoptosis claim, we just listed Annexin-V and considered it sufficient. That approached kept our list simple and concise. We detailed our methodology it a bit more in materials and methods section to clarify this.

However, if the claim specifically states MTT without mentioning further experimentation we focused on that. For example: article no4 claimed proliferation rate change and performed an MTT and nucleotide incorporation assay. However, since there is this statement “MTT assay was performed to assess cell proliferation” we included it in our list as a separate entry and considered it insufficient.

Article no121 claimed viability, cytotoxicity, cell death and apoptosis. They performed MTT and Annexin-V assays. However, since they state “The cytotoxicity of mPEG–PCL micelle to MCF-7 cells was evaluated using MTT assays.” we included MTT as a separate entry in the list and considered it as insufficient.

Like this examples in all of the cases we did not focus on what really happened to the cells in the experiments, instead we focused on how it was presented/discussed in the article.

On the other hand, many articles were easier to analyze including article no9 claiming viability, cytotoxicity, apoptosis and proliferation rate inhibition. The only data they had: MTT.

We rechecked articles with multiple evidence to detect any cases in which authors provide a better follow up evidence that we might have missed. During that investigation we decided that the article no26 has stronger evidence for anti-cell activity (anti leukemic cell activity, in their case). That evidence was in tissue level and that is why we have missed it in the first place. Consequently, it was removed from the claim list. We reanalyzed the data based on this alteration and changed relevant parts accordingly.
