# Supplementary material for: From viability to cell death: Claims with insufficient evidence in high-impact cell culture studies
Source: PLoS One. 2022 Feb 22;17(2):e0250754. doi: 10.1371/journal.pone.0250754 (PMC8863264; doi:10.1371/journal.pone.0250754)
Supplement: Attachment — Submitted filename: Ozkaya 3rd response to reviewers.docx [file pone.0250754.s006.docx]

**Reviewer #3: The authors have done a good job of responding to most reviewer concerns, though on re-review I have a few limited concerns. The bulk of the data from this study is provided in an alternate web link, though I'm not sure how I feel about this. Is this a stable link? Is the journal providing certainty that this will stay active in perpetuity? This paper becomes completely illegible without these supporting data. I strongly suggest they be added as supplemental data to the manuscript itself to ensure that these data are retained.**

We have created an CSV document (excel file) and upload it as a supplementary data along with the manuscript.

**Reviewer #3: I still feel there are some lingering issues, including some raised by reviewer #4. The final question about multiple assays, I think, raises some concerns with this analysis. This addresses how articles using multiple assays or measures were treated. The author answer was that they judged the best measure and judged the article on that - though insufficient details are provided on how they judged the best assay for each specific paper and condition. Also, certainly some credit should be given to authors that couple multiple assays together - these can often provide a more complete picture of cell health than any one individual assay.**

When multiple methods are used, and different types of evidence are provided by a study we focused on the best one. Here when we say “best” what we mean is the best according to the table 1. For example, let’s say researchers have performed assays A, B and C to evaluate cytotoxicity. And let’s say, according to the table 1, A and B is “insufficient” while C is “sufficient”. In that case we just included “C” and ignored the use of “A” and “B” as they have more of a supportive role. Basically, if there were multiple types of evidence supporting a claim we only focused on “sufficient” ones, if there are any. A simple example would be apoptotic markers. Bcl and Bax expressions are usually measured along with DNA fragmentation or phosphotidyl-serine exposure. If we were to evaluate those separately, we should classify them as insufficient. But since there is a more relevant and sufficient evidence presented along with them, we exclude them from our analysis. We do agree that utilizing multiple assays would generate better evidence but we think that evaluating that would be out of scope of this work.

**Reviewer #3: I also feel like there are some interesting concerns raised by reviewer 4 in regard to controls and the author answer to it. Surely the use of an assay, without appropriate controls, is not sufficient. Yet it seems that the details of the assay and the specifics of how it was performed and controlled for are not factored into these determinations. When making this still broad statement about the credibility of these author claims, this seems an important aspect to have considered.**

Although we agree that the proper use of controls is crucial to obtain good evidence, it is again not the focus of this study. This work is planned and conducted to demonstrate the misuse and misinterpretation of some cell culture methods and its high prevalence even in high impact articles. It is not however, designed as a guide on how to design cell culture studies, perform cell culture methods, or a document about good cell culture practices. With each review we fear that the manuscript is moving away from its intended original structure and aim. Our assessments were not about the credibility of the claims, we just want to point out whether a method, if done properly, can be utilized to measure something or not. Obviously, there are many factors affecting the overall reliability of your findings including: the number of samples, how the components of the assays as well as the controls were prepared, where and how did the samples and assay components were stored, was the cell line authenticated, was the right carrier control, right blind, right positive control, right negative control used, what was the room temperature, did antibiotics affect the findings etc. We are not trying to evaluate validity of the work we investigated, and we are not trying to assess their quality. That is why we focused on the claims and methods. However, there were a few exceptions where we had to investigate the experimental design itself. All those were listed as “conditional” in the table 1. If we were to look into the details experimental design and question the experimental conditions for each, then every entry would be “conditional”, and main points we are trying to make becomes lost. However when we re-write parts of the discussion section according to the recommendations of the reviewers we included a condensed general good practices recommendations (paragraph: 293-306).

**Reviewer #4: The article revised by Ozkaya and Geyik is improved. In particular I appreciate the addition of discussion around the caveats of the study and assays discussed. However, I find that the article has gained length rather than clarity, and that the authors are still missing an opportunity to provide clear recommendations for good cell culture technique (as it pertains to all cell-based assays) and optimal assay use to address specific biological questions accurately. While I see value in drawing attention to widespread incorrect or over-interpretation from basic assays, the potential for impact lies in being explicit about what would have been needed for a claim to be deemed sufficient. This information is only included in the Methods and is incomplete (examples are provided), the authors could consider adding a summary table of assays and the conclusions that can be drawn from them. Although I will leave it to the editor to decide whether or not this falls within the scope of the current paper.**

We just want to point out again that the aim of this work is not to provide readers with recommendations for good cell culture technique. Although we agree that the article has gained length rather than clarity, it is mostly because the reviewers wanted to turn this work into something we never intended it to be. Consequently, with each revision, we are just trying to change the manuscript based on the recommendations without altering the core. And the core is just about drawing attention to widespread incorrect or over-interpretation of some basic assays. We are very grateful for the invaluable recommendations, and it is really a pleasure to see that there are researchers out there as enthusiastic as we are about in-vitro practices. However, we are neither ready nor willing to turn this work into a guide on good cell culture practices.

We believe that the most important part of a scientific paper is the methods section. Placing the information regarding our approach on that section was a deliberate decision. We believe that methods part must contain all the information required to understand what was done in a study. Therefore, we placed both definitions we embraced along with our strategy to evaluate the claims in that section. The reviewer considers this information as incomplete because it does not include information regarding how to conduct and correctly interpret each method. However, we do not want to include those in this study. However, we did include some recommendations (paragraph: 293-306)

**Reviewer #4: I continue to disagree with the authors’ assessment that ‘viability measurement is the only purpose of an MTT assay that is somewhat acceptable’, when really it is evaluating changes in metabolic activity in a population of cells. The same number of cells can have vastly different MTT readouts based on treatment, and while I agree that using these assays to assert cell death is particularly problematic, the oversimplification is detrimental to the paper. An increase in signal does not always indicate an increase in cell number.**

We have made our points on this in the previous revision. We would like to leave the final decision on this to the editor.

**Reviewer #4: Remove references to the discussion from the Methods section.**

References were removed.

**Reviewer #4: I do not understand why cell count and DNA content are only considered sufficient evidence for proliferation in the absence of treatment. Background levels of cell death need to be assayed under all conditions, controls included.**

We agree with the necessity of evaluating the basal level of death. However, studies we investigated did not measure background cell death when they claim proliferation. That is why we considered only the absence of treatment where the effects of cell death on the measurement is minimal, as acceptable.

**Reviewer #4 Why is ‘Real-time cell imaging’ (which I assume to refer to time-lapse microscopy) insufficient for quantifying cell death? Watching cells over time is both a straightforward and accurate way to monitor cell fate.**

There was just one study where the “real-time cell imaging” was used to evaluate cell death (#49). In that study researchers just counted the number of viable cells over time. The graph was presented as “%viability” but they refer the data as an indicator of cell death.

**Reviewer #4 DAPI is membrane permeable, how is it correctly used to test membrane integrity? It can be used for DNA content, and typically is used to complement a membrane impermeable dye to accurately evaluate the fraction of cell death.**

DAPI can be membrane-permeable depending on the concentration and the incubation period but its rate of absorption by the cells is greatly increased in non-viable cells and fixated cells compared to viable ones. Therefore, it can be used to measure membrane integrity.

1. <https://www.ncbi.nlm.nih.gov/pmc/articles/PMC4496231/>
2. <https://pubmed.ncbi.nlm.nih.gov/27037070/>

**Reviewer #4 Same concern for acridine orange and Hoechst in the Viability section of the table. Unless they are used with GhostDye (or PI etc), or DNA content is accounted for, they can only yield overall cell counts.**

In all the cases, acridine orange and Hoechst were used along with an indicator of cell death:

- Hoechst with PI (#60)
- Acridine orange with EtBr (#68)
- Acridine orange with PI (#77)
- Acridine orange with PI (#77)
- Acridine orange with DAPI (#77)

**Reviewer #4 Please clarify the difference between ‘Dye inclusion’ and ‘Membrane integrity’.**

Dye inclusion: methods utilizing dyes which are taken up by and indicate viable cells. Viable cell dyes.

Membrane integrity: methods utilizing dyes which can indicate the loss of membrane integrity. Non-viable cell dyes.

**Reviewer #4 Propidium iodine should be Propidium iodide.**

Corrected

**Reviewer #4 pH3 positive cells represent a small fraction of a population and can represent cells arrested in M-phase, their presence alone does not reflect the overall proliferative potential of a population.**

In the study (#95) researchers accepted “lack of pH3 positive cells” as an indicator of halted proliferation. The logic is: “if there are no cells in M-phase in a group of cells, it means that the population is not proliferating.”. That is why we considered their evidence as sufficient. We included this explanation in Table 1.

**Reviewer #4 Change in proliferation is not limited to decrease in rate, perturbations can also stimulate proliferation.**

We were not trying to claim that the change to be limited to the decrease. We have made changes in the manuscript to clarify this.

**Reviewer #4 The discussion needs to be re-worked and copy edited. In particular lines 297-310. The discussion of IC50 values does not seem to fit – although these methods aim to improve reproducibility of large-scale drug response studies, this is somewhat separate from the issues the current paper is trying to address.**

That paragraph almost entirely consists of sentences added as a response to the previous criticism provided by the reviewers. We agree that it is disconnected. We have re-written the part as suggested. IC50 part was removed.
